# Impact of Robotic-Assisted Gait Therapy on Depression and Anxiety Symptoms in Patients with Subacute Spinal Cord Injuries (SCIs)—A Prospective Clinical Study

**DOI:** 10.3390/jcm12227153

**Published:** 2023-11-17

**Authors:** Alicja Widuch-Spodyniuk, Beata Tarnacka, Bogumił Korczyński, Justyna Wiśniowska

**Affiliations:** 1 Research Institute for Innovative Methods of Rehabilitation of Patients with Spinal Cord Injury in Kamien Pomorski, Health Resort Kamien Pomorski, 72-400 Kamień Pomorski, Poland; alicjamariawiduch@gmail.com (A.W.-S.);; 2 Department of Rehabilitation, Medical University of Warsaw, 02-091 Warsaw, Poland; 3 Department of Rehabilitation, Eleonora Reicher National Institute of Geriatrics, Rheumatology and Rehabilitation, 02-637 Warsaw, Poland; justyna.wisniowska@spartanska.pl

**Keywords:** spinal cord injury, robotic rehabilitation, coordinative rehabilitation, depression, mood, anxiety

## Abstract

Background: Mood disorders, especially depression, and emotional difficulties such as anxiety are very common problems among patients with spinal cord injuries (SCIs). The lack of physical training may deteriorate their mental state, which, in turn, has a significant impact on their improvement in functioning. The aim of the present study was to examine the influence of innovative rehabilitation approaches involving robotic-assisted gait therapy (RAGT) on the depression and anxiety symptoms in patients with SCI. Methods: A total of 110 participants with subacute SCIs were enrolled in this single-center, single-blinded, single-arm, prospective study; patients were divided into experimental (robotic-assisted gait therapy (RAGT)) and control (conventional gait therapy with dynamic parapodium (DPT)) groups. They received five training sessions per week over 7 weeks. At the beginning and end of therapy, the severity of depression was assessed via the Depression Assessment Questionnaire (KPD), and that of anxiety symptoms was assessed via the State–Trait Anxiety Inventory (STAI X-1). Results: SCI patients in both groups experienced significantly lower levels of anxiety- and depression-related symptoms after completing the seven-week rehabilitation program (KPD: Z = 6.35, *p* < 0.001, r = 0.43; STAI X-1: Z = −6.20, *p* < 0.001, r = 0.42). In the RAGT group, post-rehabilitation measurements also indicated an improvement in psychological functioning (i.e., decreases in depression and anxiety and an increase in self-regulation (SR)). Significant results were noted for each variable (STAI X-1: Z = −4.93; KPD: Z = −5.26; SR: Z = −3.21). In the control group, there were also decreases in the effects on depression and state anxiety and an increase in self-regulation ability (STAI X-1: Z = −4.01; KPD: Z = −3.65; SR: Z = −2.83). The rehabilitation modality did not appear to have a statistically significant relationship with the magnitude of improvement in the Depression Assessment Questionnaire (KPD) (including self-regulation) and State–Trait Anxiety Inventory (STAI) scores. However, there were some significant differences when comparing the groups by the extent and depth of the injury and type of paralysis. Moreover, the study did not find any significant relationships between improvements in physical aspects and changes in psychological factors. Conclusions: Subjects in the robotic-assisted gait therapy (RAGD) and dynamic parapodium training (DPT) groups experienced decreases in anxiety and depression after a 7-week rehabilitation program. However, the rehabilitation modality (DPT vs. RAGT) did not differentiate between the patients with spinal cord injuries in terms of the magnitude of this change. Our results suggest that individuals with severe neurological conditions and complete spinal cord injuries (AIS A, according to the Abbreviated Injury Scale classification) may experience greater benefits in terms of changes in the psychological parameters after rehabilitation with RAGT.

## 1. Introduction

There are approximately 10.5 new traumatic spinal cord injury (SCI) diagnoses per 100,000 people worldwide each year [1]. Based on epidemiological data for Poland, the majority of people in this population are males (the sex ratio is 2.5 females to 6 males), the average age is >40 years and the most common cause of injury is traffic accidents [2,3]. Spinal cord injuries directly lead to losses of or limitations to the motor and sensory functions at the level of injury and below. Secondary symptoms are usually sphincter dysfunction, chronic neuropathic pain, autonomic dysreflexia, respiratory failure, sexual dysfunction, digestive disorders and respiratory failure [4]. Direct and secondary somatic symptoms after SCI, as well as the loss of independence and the need to reorganize functioning in family, social and professional life, can affect the deterioration in the psychological well-being, including quality of life, body image (attitudes toward the body at the level of thoughts, emotions and behavior), self-confidence, belief in their own abilities and perception of their social attractiveness [5,6,7,8,9]. The most-frequently described psychiatric symptoms are anxiety, lowered mood and clinical depression. The second most described mental problems are anxiety disorders, including generalized anxiety disorder (GAD) and post-traumatic stress disorder (PTSD). It is estimated to affect approximately 15–32% of people with SCIs, depending on the measure used [10]. The risk rate for depression among SCI patients undergoing inpatient treatment ranges between 20 and 43%, and the average of the results obtained from different studies is 30% [11]; therefore, the prevalence of anxiety and depression among people with spinal cord injuries is higher than in the general population [11]. 

Rehabilitation, other regular exercise and physical activity significantly reduced stress and depression-related symptoms, and improved quality of life and social interactions [12,13]. They may also have preventive effects on the quality-of-life declines after a spinal injury, reducing pain and increasing sense of control, fitness and performance [12,13]. The endocannabinoid system (i.e., its components, pathways and ligands) may have preventive and therapeutic effects on neurological impairment and neurodegenerative diseases, as well as on mood and affect [14,15]. 2-Arachidonoylglycerol (2-AG) signaling to this system has an important role in adaptation to stress, anxiety, and depressive behavior (Sibers, Balchin, Badse). 2-AG is a full agonist of cannabinoid receptors (CB1 and CB2) and acts as a retrograde eCB signaling molecule [14,16]. Isometric exercise, prolonged and regular physical activity have been shown to increase the serum levels of the Endocannabinoids (eCBs) anandamide (AEA) and (2-AG) [14,15,16,17,18].

Due to the breadth and diversity of health problems after SCI, early, intensive and, above all, multispecialty assistance is essential. An early coordinated rehabilitation program makes it possible to anticipate its implications in terms of somatic and psychological health in the form of reduced anxiety and depression [19,20,21].

One of the most important and desirable goals of rehabilitation after SCI (regardless of the level, depth of the injury, time since the injury, and age of the patient) is to achieve the ability to maintain an upright position and regain the gait function [22,23]. Patients after SCI use orthoses or wheelchairs in order to transfer from one place to another. Standing and walking bring a lot of benefits to SCI patients, such as prevention of pressure sores, cardiovascular events, decreasing bone osteoporosis and improving the function of the digestive system. The upright position not only has an effect on secondary physical symptoms, but it is also related to improved quality of life, self-confidence and a sense of independence [24,25]. Conventional SCI rehabilitation (in terms of uprighting and gait re-education) involves dynamic parapodium therapy (DPT) [26]. Parapodium is a device that supports gait therapy and allows the patient to achieve an upright position or gait training. The patient’s movement consists of a pendulum motion (from side to side), which deviates significantly from the correct gait pattern [27]. The robotic-assisted gait therapy (RAGT) is mostly used for lower-extremity and body-weight-supported treadmill training. It offers a number of advantages, including the ability to start therapy early after an injury, and it is therapeutic for people with significant lower limb paralysis. In addition, it allows for the adjustment of parameters such as the speed, weight-bearing relief (intensity) and length of the training session to suit the patient’s current capabilities, and it allows for the accurate monitoring of the patient’s progress. Robotic-assisted gait training uses stationary devices, such as the Lokomat device and exoskeletons. The U.S. Food and Drug Administration defines a powered exoskeleton as follows: it “(…) is a prescription device that is composed of an external, powered, motorized orthosis that is placed over a person’s paralyzed or weakened lower extremity limb(s) for medical purposes” [28]. There are two groups of exoskeletons: assistive exoskeletons and rehabilitation exoskeletons. A rehabilitation exoskeleton is a device that provides support for the patient’s total body weight [29]. It is an equally promising, safe and well-tolerated method of therapy for patients with neurological disorders, including post-spinal-cord-injury (SCI) patients [30,31,32,33]. Modern multifaceted rehabilitation using RAGT not only has a beneficial effect on improving the sensorimotor, kinematic and autonomic functions, but it also allows for a greater sense of control over one’s own body and independence, thereby contributing to an improvement in psychological functioning and the subjective assessment of one’s quality of life [5,34,35,36,37,38,39,40,41].

During this therapy, metabolic energy expenditure and fatiguability in paraplegic persons is much more pronounced in comparison with RAGT. The walking speed of an SCI patient with such orthosis is significantly less than that of normal walking, or RAGT therapy. Robotic therapy allows for a much greater number of steps. In most gait orthosis, the high value of the force is transmitted to the upper limb joints, which can increase shoulder pain incidence. Although dynamic parapodium training DPT is widely used, there is only one study that has compared rehabilitation with DPT versus rehabilitation with RAGT. The results of the study showed that both DPT and RAGT rehabilitation can improve the gait function (WISCI II) and muscle strength (MS). However, patients with RAGT therapy achieved significantly higher scores for the above parameters [26]. Rehabilitation robots are increasingly being used to rehabilitate people with spinal cord injuries. Conventional SCI rehabilitation (in terms of uprighting and gait re-education) uses training with a dynamic parapodium [26]. The dynamic parapodium is a device that allows the patient to achieve an upright position while still allowing them to move. However, the dynamic parapodium has its limitations. The patient’s movement consists of a pendulum motion (from side to side), which deviates significantly from the correct gait pattern [27].

There are few studies describing rehabilitation using RAGT that include mental status. There have been no previous studies in the literature comparing the effects of RAGT vs. DPT therapy on psychological aspects. Moreover, most of previous studies focused on quality of life rather than emotional difficulties or affective disorders, or they include people with other neurological disorders, such as multiple sclerosis or stroke [42,43,44,45,46,47,48]. The results of these trials are often inconclusive. Some studies suggest an association between RAGT and a reduction in depressive symptoms and increase in quality of life, while others have found no such association. Others emphasize its relevance to and equivalence with biological and social factors [33,49,50,51,52]. Relatively few studies have been conducted on the efficacy of rehabilitation with RAGT on groups of patients with spinal cord injuries; however, the analyses conducted so far are promising, especially for patients with significant hemiparesis [22,31,35,47,53,54,55,56,57,58,59]. The researchers emphasize that RAGT should not replace conventional rehabilitation but complement it [49,50,60,61,62]. The rehabilitation procedures have also not been delineated in terms of the duration, frequency and intensity of training, among others [63].

In addition, we set out to see if patients with paraplegia present different levels of anxiety and depression than patients with tetraplegia in patients from the experimental and control groups. Simultaneously, the type of rehabilitation is significant in terms of changes in the symptoms of depression and state anxiety in patients with different degrees of injury on the Abbreviated Injury Scale (AIS) (AIS A vs. AIS B, C, D). Patients with spinal cord injury differ significantly in their neurological status, which has a major impact on their potential abilities and functional status [64,65,66]. In this study, we decided to divided patients into separate groups according to the AIS classification (AIS A with complete injury and AIS B, C, D with incomplete injury) and the type of paralysis (tetraplegia and paraplegia).

All study patients received psychological support, including cognitive behavioral therapy (CBT). CBT therapy has been scientifically proven to be effective in changing maladaptive thinking and behavioral patterns that contribute to people’s psychological problems [67].

In this study, we assumed the following hypotheses:

1. Both the rehabilitation group with DPT (S0) and the rehabilitation group with RAGT (S1) will show significant improvements in the severity of symptoms related to depression and state anxiety;
2. Patients in the experimental group (S1) will achieve greater improvements in the psychological indicators (severity of depression-related symptoms and state anxiety) compared to those in the control group (S0);
3. The improvement in the psychological parameters (i.e., the decrease in symptoms related to depression and state anxiety) will have a significant relationship with the improvement in the gait function and functional independence after SCI.

Based on the available research, we hypothesized that SCI patients would experience less anxiety and lower levels of depression-related symptoms after both forms of therapy, but that they would do so to a greater extent after RAGT [5,12,13,19,20,41,62,68]. The main objective of our study was to analyze the association of RAGT rehabilitation with symptoms related to depression and anxiety, as well as with clinical status. In addition, we assessed whether the level and extent of the injury and the type of paralysis affect the magnitude of changes in the symptom levels of depression and anxiety (the condition after the 7-week rehabilitation program), and whether the changes in these parameters are related to improvements in the medical parameters (i.e., functional independence and mobility and the extent of the recovery of the gait function).

## 2. Materials and Methods

### 2.1. Study Protocol

This study was a single-blinded, prospective, clinical study. The study was conducted according to the Declaration of Helsinki committee and obtained approval from the Ethical Board of the District Medical Chamber in Szczecin, Poland (Nr OIL-Sz/MF/KB/452/05/07/2018; Nr OIL-SZ/MF/KB/450/UKP/10/2018). Before being accepted into the study, all participants underwent medical examinations conducted by physicians specialized in neurological physical rehabilitation and medicine, physiotherapists and a psychologist.

Medical inclusion criteria included the following: time since injury ranging from 3 months to 2 years; the general condition of the patient defined as conscious, able to cooperate with the physiotherapist and adapted to the upright position; complete and incomplete SCIs (cervical, thoracic or lumbar) with preserved flexion and extension functions at the elbow and wrist; surgical stabilization of the spine in the phase of completed bone fusion; no contraindications to rehabilitation arising from, among other things, venous thrombosis, pulmonary embolism, orthostatic hypotension, epilepsy or infection; body weight not exceeding 120 kg; height between 150 and 190 cm. Exclusion criteria included the following: high and complete tetraplegia and very low lumbar spine injury; lack of completion of bone fusion after established spinal stabilization; lack of completed bone fusion after spine surgery; respiratory insufficiency, circulatory insufficiency III and New York Heart Association (NYHA) class IV; osteoporosis; lower-limb shortening of more than 2 cm; the presence of decubitus ulcers, deep abrasions or skin lesions that could be exacerbated by the robotic system; intensive spasticity (4 points on the Ashworth scale) and muscle contractures that make it impossible to conduct robotic rehabilitation; past or present neurological disorders (i.e., traumatic spinal stroke, multiple sclerosis, childhood cerebral palsy); symptoms of recurrent autonomic dysreflexia. In the psychological studies, additional exclusion criteria were the finding of a reduced general level of intellectual functioning preventing the completion of the questionnaire tests and an age below 16 years (there is no normalization of the psychological tools used for this age group).

All patients underwent two physiotherapeutic, medical and psychological assessments each by professionals unfamiliar with the purpose of the study at the beginning and end of the 7-week treatment program. Research tools with proven validity and reliability were used to verify the hypotheses. The research procedure followed scheme No. 1 each time. Medical, physical and neurological examination included medical history and questionnaire tests using the Spinal Cord Independence Measure, version III (SCIM-III), and the Walking Index for Spinal Cord Injury, version II (WISCI-II). The test procedure was carried out using The American Spinal Cord Injury Association (ASIA) Impairment Scale (AIS). The psychological examination included a structured interview and the administration of surveys using questionnaires (including the Depression Assessment Questionnaire (KPD) and State–Trait Anxiety Inventory (STAI X-1)). To standardize the research procedure, the psychologist read the questionnaire questions and marked the answers. This was necessary due to the motor difficulties of some patients (i.e., problems marking answers or turning pages).

### 2.2. Physiotherapy and Psychological Intervention

The rehabilitation program consisted of two phases lasting three weeks each, with a one-week break in between. Rehabilitation interventions took place six days a week. Psychological activities were held once a week and included individual sessions (targeting the patients’ needs and difficulties based on their resources) and group therapy.

Patients were enrolled in two groups by coin toss: a control group (S0) subjected to conventional gait therapy with DPT, and an experimental group subjected to rehabilitation using RAGT. The mentioned therapies lasted 30 min each. Patients enrolled in the experimental group (S1) received RAGT sessions using the EKSO-GT exoskeleton (model EKSO 1 by Ekso Bionics, San Rafael, CA, USA; year of manufacture: 2014) or Lokomat Pro (model LO218 by Hocoma AG, Volketswil, Switzerland; year of manufacture: 2014) in addition to the general standard physiotherapy training program based on proprioceptive neuromuscular facilitation. All participants from the Lokomat group with incomplete SCIs started with 60% body weight support and an initial treadmill speed of 1.5 km/h. Patients with complete SCIs started with 90–100% body weight support. In patients with the EKSO-GT, a minimum of 100 steps was required per session for DPT 20. All patients in both the S0 and S1 groups received psychological support; meetings were held three times during the cycle of the seven-week rehabilitation program, and 50-min individual sessions based on cognitive–behavioral-therapy (CBT) techniques were held at least once a week (at least eight times during the course of the camp). In general, CBT therapy is aimed at changing maladaptive patterns of thinking and behavior [67]. The program and purpose of the meetings were tailored to each patient’s needs and problems and were closely coordinated with the patient. In addition to individual meetings, weekly 90-min group therapy meetings were conducted based on the CBT method rational behavior therapy using motivational interviewing and mindfulness-based cognitive therapy. The group therapy was aimed at the general difficulties faced by people after SCI, as well as at exchanging information, supporting other patients and receiving feedback from them.

### 2.3. Participants

Patients self-reported from all over the country to participate in the study. They agreed to participate and signed an informed consent form before the study. Based on the criteria, three patients did not qualify for the psychological testing. One of the subjects had moderate intellectual disabilities and two of them were under the age of 16 years (scheme No. 1).

A simple randomization method was used to assign patients to comparison groups. The random assignment to groups was performed by a medical staff member, unaware of the study’s purpose (a blinded investigator). The medical team, paramedical team and psychologist were unaware of the purpose of the study when performing the treatment and therapeutic measures (single blinding).

A total of 110 patients completed the study; 79 of them were assigned to the S1 group participating in rehabilitation with RAGT, including 62 men and 17 women, and 31 were assigned to the S0 group, including 27 men and 4 women. The disproportion in the number of control group patients was caused by patients resigning when they were not assigned to RAGD rehabilitation group (see Table 1). Also, the situation caused by the COVID-19 pandemic caused 11 patients in the S0 group to not complete the rehabilitation cycle.

Comparisons of the S0 and S1 groups with sociodemographic and other characteristic variables showed that there were no differences between the groups at the initial level. In both groups, the majority were men and people with higher education, in formal relationships. The largest group was made up of respondents living in large cities. The most common cause of injury was a fall from a height of more than 1 m. The main level of the neurological damage was Th. The median ages were 37 (S0) and 36 (S1) years, and the median times since the accident, respectively, were 13 and 12 months.

**Figure 1:**
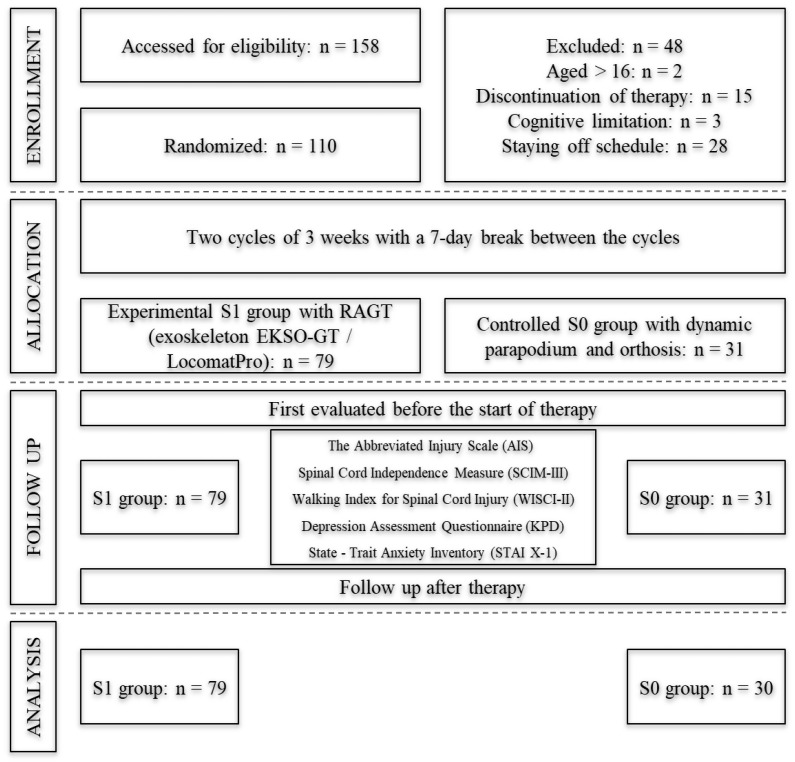
Flowchart of details of patients’ recruitment.

The study flowchart is shown in Figure 1.

#### 2.3.1. Primary Outcome Measures

In this analysis, the primary outcome measures were the overall depression severity scores and the anxiety severity status measure, while the second-order indicators were the individual depression symptom severity scores, the scores for the level of functional independence and mobility after SCI and the gait function measure.

To estimate the severity of the depression symptoms, the Depression Assessment Questionnaire (KPD) by E. Łojek, J. Stańczak, A. Wójcik was used. The KPD measures the severity of depression and its individual symptoms and includes an additional scale to assess the subjects’ self-regulatory abilities. Its theoretical basis is based on data on the symptoms and mechanisms of depression in accordance with international diagnostic criteria. The overall score (WO) of the KPD indicates the general severity of the depression. Possible scores range from 60 (no depression) to 240 (severe depression). A WO of 130 points is the cutoff between a normal score and a score suggesting depressive disorders [69].

To measure anxiety as a state, the STAI X-1 by C.D. Spielberger, R.L. Gorsuch and R.E. Lushene was used. State anxiety is understood as tension related to a current situation. Possible scores range from 20 (low anxiety) to 80 (high anxiety) [70].

#### 2.3.2. Secondary Outcome Measures

Additional scales of the KPD were used to measure the severity of individual depressive symptoms. The KPD consists of five scales: DPUE—Cognitive deficits and energy loss (refers to symptoms associated with decreased learning performance and impaired attention and memory performance, as well as deterioration in psychomotor functions and loss of energy; the range of scores is from 19 to 76 points); MSPA—Thinking about death, pessimism and alienation (refers to the measurement of the severity of the loss of meaning in life, social withdrawal and emotions with negative signs; the lowest possible raw score is 15 and the highest is 60, indicating the very high severity of the described symptoms); PWNL—Guilt and anxiety tension (measures the severity of fear, anxiety and related emotional tension and the tendency to blame oneself for various situations, behaviors and thoughts, and it also allows for an estimation of one’s level of self-esteem and sense of social attractiveness; the score range is from 16 to 64); OPSZ—Psychosomatic symptoms and decline in interest (refers to the subjective assessment of one’s somatic and mental health, and it also allows for a determination of the decrease in previous interests (anhedonia); the range of raw scores is from 10 to 40); SR—Self-regulation (an additional scale used to measure a subject’s emotional and cognitive resources protecting them from depression; the minimum number of points possible is 15 and the maximum is 60). The sum of the scores of the first four scales affects the overall score of the severity of the symptoms associated with depression (WO) [69].

In order to diagnose and assess the neurological and functional statuses, tests were conducted using the following measures: The AIS, which was used to assess the motor and sensory functions; we divided patients according to the AIS to A, B, C, D or E subgroups, where AIS A refers to complete SCI and AIS B, C and D refer to incomplete SCI [71]. The Walking Index for Spinal Cord Injury (WISCI-II), which was used to measure the improvement in the gait function of our patients after SCI. It is the most sensitive measure in terms of changes in the gait ability compared to other leading measurement scales [72]. The index provides an assessment of the level of the gait function. A level of 0 indicates the inability to stand and participate in walking, and a level of 21 indicates the ability to walk without the use of aids, orthoses or assistance from others [73]. The Spinal Cord Independence Measure, version III (SCIM-III), was used for functional assessment because it is a reliable and accurate scale for assessing skills in the daily functioning of patients with spinal cord injuries, with proven adequacy in group studies [74], and it is the only comprehensive scale for assessing the ability of patients to perform basic daily activities. Version III contains 19 tasks divided into three areas: self-care; breathing and sphincter control; mobility. The total score ranges from 0 (indicating total dependence) to 100 (indicating total independence) [75].

### 2.4. Statistical Analyses

In this study, continuous data are presented as mean values and standard deviations (SDs) and categorical data are presented as percentages. Distributions were checked, and descriptive statistics of the quantitative variables were calculated. The normality of the distributions was tested with the Shapiro–Wilk test, but the skewness and kurtosis values were also considered. Due to the violation of the assumptions of normal distribution and numerous outlier observations exceeding the third standard deviation, analyses were based on non-parametric tests. Comparisons of the nominal and ordinal variables for the group characteristics were based on chi-square tests of independence along with Fisher’s exact test. In addition, correlation analyses with Spearman’s rho coefficient were performed to assess the relationships between the variables. In order to determine the effect size used to measure the impact of the strength of the relationship between the independent variables studied and dependent variables, we adopted cutoff values at significance levels of *p* < 0.001, *p* < 0.01 and *p* < 0.05. The effect size results were determined and interpreted as correlation values: 0.00–0.19: “very weak”; 0.20–0.39: “weak”; 0.40–0.59: “moderate”; 0.60–0.79: “strong”; 0.80–1.0: “very strong” [76].

Quantitative analysis of group characteristics and normality of distribution tests were performed on a group of 110 individuals. However, further comparative analyses were performed on a group of 109 people due to missing data for one person at the second measurement in the S0 group n = 30 (see Figure 1).

Differences in the state anxiety and depression indicators between the pre-rehabilitation and post-rehabilitation measurements were tested using the Mann–Whitney U test. Similarly, this analysis was performed with the division into control and experimental groups (S0 n = 30 vs. S1 n = 79) using Wilcoxon rank-sum and signed-rank tests for this purpose. Differences in functional indices (WISCI-II and SCIM-III) were also tested separately in groups S0 and S1 and between the study groups (S0 and S1) using the Wilcoxon rank tests and Mann–Whitney U test. Next, we tested whether there were relationships between the changes that occurred between the measures of the functional indicators (WISCI-II, SCIM-III) and changes in mental health (STAI X-1, KPD). Spearman’s rho correlation analyses were performed for this purpose. Measures of anxiety and depression before and after rehabilitation were also compared, as well as changes in the anxiety and depression divided into the S0 and S1 groups and according to the AIS classification of spinal cord injury, so that four groups were compared: S0 AIS A (n = 13); S0 AIS B, C, D (n = 17); S1 AIS A (n = 30); S1 AIS B, C, D (n = 49). This analysis was then performed between subjects with two types of paralysis: paraplegia (n = 89) and tetraplegia (n = 20). These analyses were performed using a series of Wilcoxon signed-rank tests and Mann–Whitney U tests.

The present study did not perform a comparative analysis between the degree of paralysis (paraplegia vs. tetraplegia) and AIS scale scores (AIS A vs. AIS B, C, D) based on the division into study groups (S0 vs. S1) in terms of changes in the psychological parameter scores. This was due to the different sizes of the groups (S0 vs. S1; Table 1). The distribution by AIS vs. the range of paralysis was as follows: paraplegia: group S0, n = 25; group S1, n = 64; tetraplegia: group S0, n = 6; group S1, n = 15.

In summary, a total of 158 subjects qualified for our prospective, single-blind clinical trial based on specific inclusion and exclusion criteria. However, 48 people dropped out during the study (see Figure 1). A total od 110 subjects completed the study (see Table 1). However, 109 individuals were included in the analyses due to missing data. The analyses were performed using the IBM SPSS Statistics package, version 25.

The subjects underwent medical, physiotherapeutic and psychological examinations twice (at the beginning and at the end of the rehabilitation program). Statistical analysis was performed using non-parametric tests. A probability level of *p* > 0.05 was assumed.

## 3. Results

As mentioned above, 158 people qualified for the study and 110 completed it. However, 109 people were included in the statistical analyses because one person in the control group missed the second measurement (Figure 1).

In the present research, all the patients had neurological impairments after SCIs. In the S0 and S1 groups, totals of 25% and 20.3% of patients had neurological impairments at the cervical level, respectively, 48.4 and 53.2% had neurological impairments at the thoracic level, respectively, and 25.8 and 26.6% had neurological impairments at the lumbar level, respectively.

### 3.1. Severity of State Anxiety and Depression after 7-Week Rehabilitation Program

The results provided in Table 2 (the severity of the state anxiety (Z = −6.20, *p* < 0.001) and general depression (Z = 6.35, *p* < 0.001) symptoms and all the depression factors (DPUE, MSPA, PWLN, OPSZ)) were significantly decreased after the 7-week rehabilitation program, compared with the first measurement, regardless of the type of rehabilitation. The ability to self-regulate also increased significantly. The moderate effect size of rehabilitation was obtained in both the severity of depression (r = 0.42) and state anxiety (r = 0.43). Similarly, a moderate effect size in the PWLN depression factor and a weak effect size of rehabilitation in DPUE, MSPA, OPSZ and SR were obtained.

#### 3.1.1. Differences in State Anxiety, Depression and Functionality in Comparison to the Type of Rehabilitation

The results in Table 3 show significant increases in all the general depression symptoms and depression and state anxiety factors after 7 weeks of rehabilitation in both the experimental and control groups. In the RAGT group, the effect size of rehabilitation was moderate for anxiety and all the KPD subscales, except PWLN and SR. For PWLN and SR, a weak effect size of the RAGT rehabilitation was obtained.

Table 3 also shows the differences in the results in the functional scales tested between the WISCI-II and SCIM-III separately in the S0 and S1 subgroups. There was an improvement in the clinical-functioning tests in the S0 group in the second measurement compared with the first. In the S1 group, there was also a significant improvement in the patients’ clinical functioning after RAGT rehabilitation, but with greater effects on the WISCI-II scale (Z = −5.17; *p* < 0.001) and SCIM-III scale (Z = 6.99; *p* < 0.001). The effect sizes of RAGT rehabilitation on the WISCI-II (r = 0.41) and SCIM-III (r = 0.56) scales were moderate. Intergroup comparisons (S0 vs. S1) in terms of functional impairment showed a greater improvement in the S1 group, but only for the WISCI-II scale. The effect size of RAGT rehabilitation was weak. There were no statistically significant differences for the SCIM-III between the S0 and S1 groups (Z = −0.69; *p* = 0.487).

#### 3.1.2. Functional and Mental Status Correlation

Table 4 shows the relationships between the changes that occurred between the measurements in the functional indicators (WISCI-II, SCIM-III) and changes in mental health (STAI X-1, KPD). There was no significant relationship between the somatic and mental functioning changes.

### 3.2. Severity of State Anxiety and Depression according to Neurological Impairment (AIS)

The anxiety and depression comparisons before and after rehabilitation in all the patients and subgroups according to the AIS classification are provided in Table 5. The results indicate significant increases in self-regulation and decreases in anxiety and depression in both the experimental and control groups.

In the S0 and AIS A group of patients, there was a significant decrease in the state anxiety after rehabilitation with a strong effect size (Z = −3.07; *p* = 0.002; r = 0.60). However, there were no significant differences between the measurements of general depressive symptoms (Z = −1.45; *p* = 0.147). Among patients in the S0 and AIS B, C, D group, the effects sizes of DPT for the general depression score and MSPA subscale were strong. The impacts of DPT on other depression factors (DPUE, PWLN, OPSZ and SR) were moderate. In the S1 and AIS A and S1 and AIS B, C, D groups, significant decreases were also observed for each depression and state anxiety measurement. In the S1 with AIS A group, the effect size was weak for self-regulation (r = 0.26). The effect size of RAGT rehabilitation was of moderate strength for the other KPD subscales (DPUE, MSPA, PWLN, OPSZ) and STAI X-1. The S1 and AIS B, C, D group also showed statistically significant decreases in depression and anxiety symptoms between the two measures, and an increase in SR.

In addition, Table 5 presents a comparison of the AIS (AIS A vs. AIS B, C, D) groups and depression and anxiety indicators separately in the S0 and S1 groups. It turns out, in the S0 group, a greater reduction in MSPA in AIS B, C, D than in AIS A was observed (Z = −2.46; *p* = 0.014). The effect size of the DPT was moderate (r = 0.45). There were no significant differences between the two groups in the other KPD subscales (DPUE, PWLN, OPSZ, SR). In contrast, the comparison between the RAGT groups (AIS A vs. AIS B, C, D) showed that the severity of changes in anxiety and depression was quite similar. The groups did not differ in self-regulation.

### 3.3. Severity of State Anxiety and Depression according to the Type of Paralysis

The differences between the subjects with two types of paralyses (tetraplegia vs. paraplegia) for the anxiety and depression indicators are presented in Table 6.

The results indicated a significant decrease in the levels of state anxiety (Z = −5.40; *p* < 0.001; r = 0.40) and depression (Z = −5.59; *p* < 0.001; r = 0.42), including an increase in self-regulation (Z = −2.95, *p* = 0.003, r = 0.22), among people with paraplegia. The effect size on SR was weak, while the effects on the other subscales of the KPD and STAI X-1 were moderate.

In the patients with tetraplegia, there were significant decreases in the state anxiety, general depression indicators and MSPA, PWLN, OPSZ and SR subscales. Only on the MSPA subscale were there were no significant differences between the two measurements. An increase in self-regulation after the rehabilitation program was obtained (Z = −3.48; *p* < 0.001). Among those with tetraplegia, there was also a decrease in the severity of symptoms related to depression and state anxiety, and an increase in the self-regulatory capacity. The effect size was strong for SR but moderate for the other KPD and STAI subscales. The patients with tetraplegia also showed decreases in anxiety (Z = −3.08; *p* = 0.002) and the total scores for depression (Z = −3.07; *p* = 0.002), as well as in other subscales, except for the DPUE index. In this group of patients, there was also an increase in self-regulation (Z = −3.48; *p* < 0.001) after rehabilitation. The effect was strong for SR but moderate for other significant differences (i.e., general KPD scores, MSPA, PWLN and OPSZ subscales and STAI X-1).

The differences in the anxiety and depression between the groups with different types of paralyses are provided in Table 6. The results indicate that subjects with paraplegia were characterized by a smaller increase in self-regulation compared to subjects with tetraplegia. The significant differences between the groups were obtained with low size effects. No differences between the groups were observed for the other KPD subscales (DPUE, MSPA, PWLN, OPSZ) or state anxiety.

The present study did not perform a comparative analysis between the degree of paralysis (paraplegia vs. tetraplegia) and AIS scale scores (AIS A vs. AIS B, C, D) based on the division into study groups (S0 vs. S1) in terms of changes in the psychological parameter scores. This was due to the different sizes of the groups (S0 vs. S1; Table 2). The distribution by AIS vs. the range of paralysis was as follows: paraplegia: group S0, n = 25; group S1, n = 64; tetraplegia: group S0, n = 6; group S1, n = 15.

In summary, after the seven-week rehabilitation program, all patients studied achieved significant improvements in anxiety state (STAI—X1) and depression (i.e., in terms of total scores and all symptoms of depression), as well as an increase in Self-regulation (see Table 2).

Analysis of changes in psychological factors (STAI X-1, KPD) and functional status (SCIM III) and improvement in gait function (WISCI—II) after a seven-week rehabilitation program showed improvement in all parameters studied in both study groups (S0 and S1), as seen in Table 3.

Intergroup comparisons (S1 vs. S0) for functional improvement (SCIM, WISCI) showed significantly greater improvement in gait function (WISCI-II) in the S1 group compared to the S0 group. No significant between-group differences were found for SCIM-III, as seen in Table 3.

Correlation analysis showed no significant relationship between functional changes (SCIM-III and WISCI-II) and changes in psychological parameters (STAI X-1, KPD), as seen in Table 4.

Comparison of measures of psychological factors before and after rehabilitation (STAI X1, KPD) divided into four groups (i.e., S0 AIS A; S0 AIS B,C,D; S1 AIS A; S1 AIS B,C,D) showed a significant decrease in anxiety and depression, as well as an increase in Self-regulation in the groups studied, except for the S0 AIS A group, where only a significant decrease in state anxiety (STAI X-1) was observed (no differences were observed between the measures of depression, including self-regulation, in this group), as seen in Table 5.

Group comparisons according to neurological impairment (AIS A vs. AIS B, C, D) separately for each research group (S0 and S1) showed no significant differences for any of the variables tested, except for a greater change in the MSPA (Thinking about death, pessimism and alienation, KPD subscale) in the S0 group, as seen in Table 5.

An analogous analysis of the differences in psychological factors by type of paralysis (tetraplegia and paraplegia) showed that in people with tetraplegia, there was a significant decrease in anxiety and depression and all their symptoms except DPUE (Cognitive deficits and Energy loss, KPD subscale), and an increase in Self-regulation. However, in people with paraplegia, there was a significant change in every parameter tested. Intergroup comparisons did not show significant differences for state anxiety (STAI-X1) and general depression scores and its individual KPD symptoms. However, it was observed that people with tetraplegia had a significantly higher increase in Self-regulation ability compared to people with paraplegia. (See Table 6).

## 4. Discussion

### 4.1. The Impact of a 7-Week Rehabilitation Program on Functionality and Severity of Depression and State Anxiety

Our study showed significant decreases in the state anxiety and symptoms of depression and increases in self-regulation in the patients in both groups (S0 and S1) after a 7-week rehabilitation program. However, no significant difference was observed between the groups according to the type of rehabilitation (RAGT vs. DPT). Although no differences were found between the types of rehabilitation (RAGT vs. DPT) in reducing the severity of the state anxiety and symptoms of depression, it can be speculated from the results that robotic rehabilitation may be particularly important for patients with complete spinal cord injuries (AIS A). After robotic rehabilitation, a decrease in the severity of the state anxiety and a decrease in the level of perceived symptoms of depression were observed in both patients with complete SCIs and those with incomplete SCIs. In addition, we also noted no significant statistical association between the functional independence and psychological factors. A statistically significant difference was observed only for the WISCI-II scale. Patients in the rehabilitation group with RAGT achieved a greater improvement in gait function. We analyzed the association of the medical parameters with the psychological variables. We found that the improvements in the gait function (WISCI II) and functional independence (SCIM-III) were unrelated to improvements in the depression (KPD), state anxiety as a condition (STAI X-1) and self-regulation (KPD) scores.

Both, RAGT and DPT rehabilitations reduced state anxiety and depression symptoms. The mechanism for reducing depression and anxiety symptoms could be related to general fitness and performance improvement after rehabilitation. SCI patients recovered independence in everyday activities, which improved their moods and reduced their anxiety severity. In addition, rehabilitation and other regular exercise and physical activity are significantly associated with lower levels of stress and depressive symptoms, increased social participation and improvement in quality of life. They may also have a preventive effect on the decline in quality of life after spinal cord injury, primarily by reducing the patient’s pain levels, increasing their feelings of control and mastery and improving their fitness and performance In addition, as mentioned in the introduction, rehabilitation and other regular physical activity are significantly associated with improvement of emotional and affective states, improvement of quality of life (primarily by reducing pain levels, increasing sense of control and mastery, improving fitness and efficiency and increasing participation in social life) [12,13]. It can also be assumed that improvement of anxiety and symptoms of depression is a natural process of recovery and adaptation to disability [77]. In addition, the time since injury and adaptation to new living conditions through the introduction of practical training, such as the independent use of handicapped accessible cars, improving wheelchair mobility, etc., may have a positive impact on the emotional and affective state of spinal cord injury patients.

It can also be assumed that the improvement in the psychological parameters of both study groups may have been influenced by their therapeutic interactions with CBT. This hypothesis appears to be supported by the results of studies and meta-analyses that show improvements in, among others, anxiety and depression in people with spinal cord injuries following CBT [78,79,80].

The present study did not show an association between improvements in the gait function and functional independence after SCI and symptoms of anxiety and depression. These aspects are not correlated, which may be partly explained by the patients’ subjective assessments of their health. The perceptions of health and objective physical-condition functional assessments were not the same among the SCI participants.

In opposite to our findings, the results of a review and meta-analysis by den Brave, M., et al. (2023) indicated improvement in depression-related outcomes after RAGT training [68]. Our results are also different from those obtained by Shahin et al. (2017), who noted a statistically significant difference between subjects rehabilitated with RAGT and those rehabilitated using conventional methods with DPT. This discrepancy may be due to the use of a different measurement tool and the size of the study group [81]. The referenced study was conducted on a group of forty people (N = 40) and depression was measured using the Beck Depression Inventory. However, other meta-analyses and retrospective studies indicate that there is insufficient evidence of an association between robotic rehabilitation and reductions in depression or improvements in quality of life [47]. Nevertheless, our results are largely consistent with other studies indicating that all rehabilitation interactions have beneficial effects on psychological well-being, emotional state and mood, and on lowering depressive symptoms. Many researchers emphasize that the most optimal results in re-educating gait and improving the overall functioning of patients with SCIs are obtained with conventional rehabilitation and the coordinated interaction of multiple specialist combinations [65,81]. This is due, among other things, to the peculiarities of neurological disorders, the physical effects of which are multi-systemic, translating into psycho-sociological aspects.

### 4.2. State Anxiety/Depression Status and Neurological Impairment, Depth of Injury and Type of Paralysis

Patients who participated in rehabilitation with the dynamic parapodium (S0) showed statistically significant reductions only in the level of state anxiety, while no improvements in the symptoms of depression were observed in patients with complete SCIs (AIS A). As we emphasized above, patients rehabilitated with RAGT with complete SCIs (AIS A) and incomplete impairment (AIS B, C, D) experienced reductions in both state anxiety and symptoms of depression, in contrast to patients with complete impairment (AIS A), who reported only reductions in state anxiety.

In the present study, there was also a significantly greater decrease in symptoms related to thinking about death and feeling alienated (moderate effect) in the control group among patients with incomplete spinal cord injuries compared with those with complete injuries (AIS A) according to the AIS A classification. Perhaps this can be explained by their improved neurological condition, which is important for greater mobility and independence. This allows patients to participate more in social life and feel higher levels of hope and satisfaction with life, and to make plans for the future.

Additionally, we observed no significant differences between the groups by type of paralysis (paraplegia vs. tetraplegia) in the reductions in state anxiety and symptoms of depression. The only difference was in the increase in self-regulation when comparing improvements by type of paralysis (i.e., paraplegia vs. tetraplegia). The results showed a greater increase in self-regulation in people with tetraplegia. The higher increase in the self-regulation abilities of people with tetraplegia can perhaps be explained by the initial low expectation of being able to change their condition and the low confidence in their ability to achieve their rehabilitation goals. Medical and paramedical staff are geared towards providing positive reinforcement, jointly setting motivating and satisfactory but realistic rehabilitation goals and giving patients the hope of achieving them, which positively influences the patients’ growth in self-confidence and self-control and their subjective belief in their own self-efficacy in coping [72]. Hopes for the coordinated rehabilitation program allow us to anticipate its implications in the psychological dimension in the form of reductions in state anxiety and depression (i.e., common symptoms co-occurring with spinal cord injury) [21]. Psychological support and assistance are, according to many studies, as important as rehabilitation in the quest to improve the quality of life of people with spinal cord injuries [82].

### 4.3. General Discussion

Our study showed that all the study patients experienced fewer anxiety- and depression-related symptoms after the 7-week rehabilitation program. However, we did not observe significant differences between the groups (DPT vs. RAGT). The data obtained allow us to assume that RAGT can be recommended to patients with severe neurological conditions and total spinal cord injuries within the context of their emotional and affective states. Our research also suggests that an individual’s perception of their own health and their actual physical health may not be the same. We can also assume that psychological support during rehabilitation is an essential part of the process.

There are several limitations to this study: (1) First of all, a sample size was not estimated before the start of the study. Despite this, a very large group of SCI patients participated in study. (2) Patients were self-recruited, which may have falsified the representativeness of this group among the general population of people with SCIs. (3) There were disproportionate numbers of patients between the experimental and control groups, and between those with complete and incomplete SCIs. This was mainly due to the decision of many patients to withdraw from the rehabilitation program after being informed that they had been assigned to the control group (DPT). Many patients hoped to have the opportunity to participate in robotic therapy. (4) There was a lack of qualitative control for and quantitative measurement of the patient-reported fear of falling that was experienced during exoskeleton rehabilitation. (5) Due to the patients’ motor disabilities, the psychologist read the questionnaire questions, which may have disturbed the objectivity of the survey results. (6) The strict inclusion and exclusion criteria may have limited the reliability of generalizing the results to the general population. (7) It is also worth mentioning that non-parametric tests were used in this analysis, although the power of these tests (calculated as 1 minus the probability of making a 2nd degree error: an error of the second type, which involves accepting a null hypothesis that is in fact false. The probability of making an error of the second type is denoted by the symbol β. The values of α (the significance level) and β are related in such a way that a decrease in the probability of α causes an increase in the probability of β) is lower than for parametric tests. However, in some cases, their use is a better or even necessary choice. In the present study, the quantitative variables had a non-normal distribution, and the averages in the study groups were not equal or similar.

Future research should pay attention to the adaptation of the tools that measure mental health status to the special needs and difficulties of SCI patients or consider the validation of the existing instruments in this group of patients. Important research issues related to people with spinal cord injuries that should be considered in future studies are as follows: sexual problems; chronic pain; spastic tension vs. neuroticism and anxiety; the relationship of motivation levels on the outcomes of rehabilitation progress, anxiety and depression among caregivers and families of people with SCI; the social adaptation and socioeconomic functioning of patients post-institutional rehabilitation.

## 5. Conclusions

In conclusion, both types of rehabilitation (RAGT and DPT) appeared to be associated with reductions in anxiety- and depression-related symptoms. Rehabilitation with RAGT contributes to the strengthening the self-regulation abilities of patients with tetraplegia.

## Figures and Tables

**Table 1:** Characteristics of the study group.

Group	S0 (N = 31; 28.2%)	S1 (N = 79; 71.8%)	*p*-Value
**Sex**			
Women	4 (12.9%)	17 (21.5%)	0.301
Men	27 (87.1%)	62 (78.5%)	
**Education**			
Enrolled in education	2 (6.5%)	4 (5.1%)	0.554 *
Elementary	0 (0.0%)	2 (2.5%)	
Vocational	6 (19.4%)	7 (8.9%)	
Secondary	6 (19.4%)	19 (24.1%)	
Higher	17 (54.8%)	47 (59.5%)	
**Accommodation**			
Countryside	8 (25.8%)	22 (28.6%)	0.912
Small town	7 (22.6%)	20 (26.0%)	
Medium-size town	6 (19.4%)	11 (14.3%)	
Big city	10 (32.3%)	24 (31.2%)	
**Marital status**			
Lack of partner	5 (16.1%)	22 (27.8%)	0.338
Informal relationship	5 (16.1%)	15 (19%)	
Formal relationship	21 (67.7%)	42 (53.2%)	
**Cause of injury**			
Vehicle accident	12 (38.7%)	24 (30.8%)	0.089 *
Fall < 1 m	2 (6.5%)	5 (6.4%)	
Fall > 1 m	8 (25.8%)	29 (37.2%)	
Dive	2 (6.5%)	2 (2.6%)	
Violence-related trauma	0 (0.0%)	1 (1.3%)	
Body crushing	4 (12.9%)	1 (1.3%)	
Others	3 (9.7%)	16 (20.5%)	
**Level of neurological impairment**			
Cervical	8 (25.8%)	16 (20.3%)	0.811
Thoracic	15 (48.4%)	42 (53.2%)	
Lumbar	8 (25.8%)	21 (26.6%)	
**Extent of spinal injury according to The Abbreviated Injury Scale (AIS)**			
AIS A	14 (45.2%)	30 (38.0%)	0.489
AIS B/C/D	17 (54.8%)	49 (62.2%)	0.666
**Type of paralysis**			
Paraplegia	25 (80.6%)	64 (81.0%)	0.965
Tetraplegia	6 (19.4%)	15 (19.0%)	
**Age**			
Median (IQR)	37.0 (22)	36 (23)	0.666
**Time from accident (months)**			
Median (IQR)	13 (13)	12 (11)	0.433

*—Fisher’s exact test.

**Table 2:** Changes in state anxiety and depression across the study sample.

Variables	The Whole Group		
	Baseline, Median (IQR)	Final Median (IQR)	Size Effect
the State–Trait Anxiety Inventory Subscale (STAI x-1)	35.00 (13.00)	31.00 (8.50)	0.42 ***
Depression Assessment Questionnaire (KPD)	89.00 (34.00)	80.50 (29.50)	0.43 ***
Cognitive deficits and energy loss and alienation (DPUE)	29.00 (10.00)	26.50 (7.50)	0.35 ***
Thinking about death, pessi mism (MSPA)	19.00 (8.00)	17.00 (5.50)	0.35 ***
Guilt and anxiety tension (PWLN)	27.00 (8.00)	24.50 (10.00)	0.43 ***
Psychosomatic symptoms and decline in interest (OPSZ)	16.00 (7.00)	13.00 (6.50)	0.32 ***
Self-regulation (SR)	46.00 (8.00)	49.00 (10.00)	0.29 ***

Abbreviations: IQR: interquartile range; size effect: r. *** *p* < 0.001.

**Table 3:** Changes in state anxiety and depression and changes in functioning rates by type of rehabilitation.

Variables	S0 (*n* = 30)			S1 (*n* = 79)			S0 (*n* = 30)	S1 (*n* = 79)	Size Effect
	Baseline, Median (IQR)	Final Median (IQR)	Size Effect	Baseline, Median (IQR)	Final Median (IQR)	Size Effect	Change Median (IQR)	Change Median (IQR)	
STAI X-1	35.50 (10.00)	31.00 (8.50)	0.52 ***	32.00 (13.00)	26.00 (5.00)	0.39 ***	5.00 (5.50)	4.00 (9.00)	0.05
KPD	90.50 (34.00)	80.50 (29.50)	0.47 ***	85.00 (30.00)	78.00 (26.00)	0.42 ***	5.50 (12.00)	6.00 (11.00)	0.04
DPUE	29.00 (10.00)	26.50 (7.50)	0.42 **	27.00 (10.00)	26.00 (8.00)	0.32 ***	2.50 (5.00)	2.00 (5.00)	0.09
MSPA	19.50 (8.00)	17.00 (5.50)	0.38 **	19.00 (5.00)	17.00 (4.00)	0.34 ***	2.00 (3.75)	1.00 (2.00)	0.07
PWLN	27.50 (9.00)	24.50 (10.00)	0.40 **	25.00 (9.00)	21.00 (8.00)	0.45 ***	11.00 (8.25)	10.00 (5.00)	0.14
OPSZ	16.00 (8.00)	13.00 (6.50)	0.40 **	15.00 (6.00)	14.00 (5.00)	0.28 ***	1.50 (4.00)	1.00 (4.00)	0.12
SR	46.00 (9.00)	49.00 (10.00)	0.37 **	46.00 (7.00)	47.00 (8.00)	0.26 **	3.00 (4.25)	2.00 (4.00)	0.11
WISCI	0.00 (4.00)	0.50 (5.75)	0.26 *	2.00 (11.00)	6.00 (15.00)	0.41 ***	0.00 (0.00)	0.00 (3.00)	0.24 *
SCIM	63.50 (27.25)	66.50 (22.25)	0.50 ***	64.00 (20.00)	70.00 (24.00)	0.56 ***	4.00 (8.25)	5.00 (7.00)	0.07

Abbreviations: S0: control group; S1: experimental group; IQR: interquartile range. The change for SR was based on the P2–P1 difference, and the changes for the other variables were based on the P1–P2 difference. Size effect: r. *** *p* < 0.001; ** *p* < 0.01; * *p* < 0.05.

**Table 4:** Correlations of functional changes (SCIM and WISCI) with changes in mental health (STAI X-1 and KPD).

Change	S0 (*n* = 30)				S1 (*n* = 79)			
	Change, STAI X-1		Change, KPD		Change, STAI		Change, KPD	
	*r_s_*	*p*	*r_s_*	*p*	*r_s_*	*p*	*r_s_*	*p*
SCIM	−0.29	0.152	−0.16	0.422	0.17	0.233	0.08	0.573
WISCI	0.03	0.902	0.04	0.841	−0.02	0.870	−0.15	0.281

**Table 5:** Changes in state anxiety and depression according to rehabilitation type and spinal cord injury classification.

**S0**	**ASIA-A (*n* = 13)**			**ASIA-B, C, D (*n* = 17)**			**ASIA-A**	**ASIA-B, C, D**	
	**Baseline,** **Median (IQR)**	**Final** **Median (IQR)**	**Size** **Effect**	**Baseline,** **Median (IQR)**	**Final** **Median (IQR)**	**Size** **Effect**	**Change,** **Median (IQR)**	**Change,** **Median (IQR)**	**Size** **Effect**
**STAI X-1**	32.00 (7.50)	27.00 (7.50)	0.60 **	38.00 (8.50)	33.00 (11.50)	0.48 **	5.00 (6.50)	5.00 (6.50)	0.07
**KPD**	78.00 (24.00)	78.00 (24.00)	0.28	97.00 (38.50)	84.00 (40.00)	0.59 ***	4.00 (10.00)	10.00 (16.50)	0.34
**DPUE**	27.00 (11.00)	24.00 (9.00)	0.37	35.00 (13.50)	28.00 (13.50)	0.47 **	2.00 (3.50)	4.00 (7.50)	0.25
**MSPA**	16.00 (5.50)	17.00 (3.50)	0.07	21.00 (13.00)	18.00 (10.50)	0.56 **	0.00 (3.50)	2.00 (4.00)	0.45 *
**PWLN**	23.00 (6.5)	24.00 (8.50)	0.37	28.00 (6.50)	25.00 (11.50)	0.44 *	11.00 (7.00)	15.00 (9.50)	0.16
**OPSZ**	16.00 (5.00)	13.00 (5.00)	0.34	19.00 (8.50)	12.00 (9.00)	0.43 *	1.00 (5.00)	2.00 (4.50)	0.05
**SR**	47.00 (5.50)	49.00 (9.00)	0.34	45.00 (11.50)	48.00 (12.50)	0.38 *	2.00 (4.50)	3.00 (7.50)	0.14
**S1**	**ASIA-A (*n* = 30)**			**ASIA-B, C, D (*n* = 49)**			**ASIA-A**	**ASIA-B, C, D**	
	**Baseline,** **Median (IQR)**	**Final** **Median (IQR)**	**Size** **Effect**	**Baseline,** **Median (IQR)**	**Final** **Median (IQR)**	**Size** **Effect**	**Change,** **Median (IQR)**	**Change,** **Median (IQR)**	**Size** **Effect**
**STAI X-1**	31.50 (11.50)	26.00 (2.75)	0.41 **	33.00 (12.00)	26.00 (6.00)	0.38 ***	4.00 (8.25)	3.00 (8.50)	<0.01
**KPD**	80.50 (33.75)	75.50 (23.50)	0.44 **	86.00 (27.00)	79.00 (31.00)	0.40 ***	6.00 (12.25)	6.00 (10.00)	<0.01
**DPUE**	26.50 (10.25)	26.00 (5.75)	0.30 **	28.00 (10.50)	26.00 (10.50)	0.33 **	2.00 (5.50)	2.00 (4.00)	0.04
**MSPA**	17.00 (5.00)	16.00 (4.00)	0.39 **	19.00 (5.50)	18.00 (4.50)	0.32 **	1.00 (2.00)	1.00 (2.00)	<0.01
**PWLN**	24.50 (7.50)	19.00 (7.50)	0.44 **	26.00 (10.00)	22.00 (10.00)	0.44 ***	9.50 (6.00)	11.00 (4.50)	0.11
**OPSZ**	14.00 (7.50)	14.00 (5.25)	0.30 **	15.00 (5.50)	14.00 (5.50)	0.27 **	1.00 (4.00)	1.00 (4.00)	0.02
**SR**	47.00 (6.00)	47.50 (9.00)	0.26 *	44.00 (8.00)	46.00 (9.50)	0.25 *	1.50 (4.25)	2.00 (4.50)	0.04

Abbreviations: S0: control group; S1: experimental group; IQR: interquartile range. The change for SR was based on the P2–P1 difference, and the changes for the other variables were based on the P1–P2 difference. Size effect: r. *** *p* < 0.001; ** *p* < 0.01; * *p* < 0.05.

**Table 6:** Changes in state anxiety and depression depending on the type of paralysis.

Variables	Paraplegia (*n* = 89)			Tetraplegia (*n* = 20)			Paraplegia (*n* = 89)	Tetraplegia (*n* = 20)	Size Effect
	Baseline, Median (IQR)	Final Median (IQR)	Size Effect	Baseline, Median (IQR)	Final Median (IQR)	Size Effect	Change Median (IQR)	Change Median (IQR)	
**STAI X-1**	33.00 (13.00)	26.00 (7.00)	0.40 ***	35.00 (13.00)	28.00 (6.75)	0.49 **	4.00 (7.00)	3.50 (8.75)	<0.01
**KPD**	86.00 (29.00)	79.00 (25.50)	0.42 ***	92.50 (36.25)	81.50 (31.00)	0.49 **	6.00 (13.00)	7.00 (8.50)	<0.01
**DPUE**	28.00 (9.50)	26.00 (7.00)	0.36 ***	26.00 (13.00)	25.00 (13.50)	0.28	2.00 (4.50)	1.50 (5.50)	0.06
**MSPA**	19.00 (5.50)	17.00 (4.00)	0.35 ***	18.50 (6.75)	18.00 (4.00)	0.32 *	1.00 (2.00)	0.50 (3.50)	0.06
**PWLN**	26.00 (8.50)	22.00 (8.50)	0.43 ***	25.50 (12.00)	23.00 (10.75)	0.47 **	10.00 (6.00)	10.50 (7.00)	0.03
**OPSZ**	15.00 (6.50)	13.00 (5.00)	0.30 ***	16.50 (7.75)	14.00 (5.50)	0.41 **	1.00 (4.00)	2.00 (3.75)	0.11
**SR**	46.00 (9.00)	47.00 (10.00)	0.22 **	46.00 (7.50)	49.00 (8.75)	0.55 ***	1.00 (4.00)	3.00 (5.50)	0.20 *

Abbreviations: IQR: interquartile range. The change for SR was based on the P2–P1 difference, and the changes in the other variables were based on the P1–P2 difference. Size effect: r. *** *p* < 0.001; ** *p* < 0.01; * *p* < 0.05.

## Data Availability

Data are contained within the article.
